# Global epidemiology of breast cancer based on risk factors: a systematic review

**DOI:** 10.3389/fonc.2023.1240098

**Published:** 2023-10-10

**Authors:** Amna Roheel, Aslam Khan, Fareeha Anwar, Zunaira Akbar, Muhammad Furqan Akhtar, Mohammad Imran Khan, Mohammad Farhan Sohail, Rizwan Ahmad

**Affiliations:** ^1^ Riphah Institute of Pharmaceutical Sciences, Riphah International University, Lahore, Islamabad, Pakistan; ^2^ Department of Natural Products, College of Clinical Pharmacy, Imam Andulrahman Bin Faisal University, Rakah, Dammam, Saudi Arabia

**Keywords:** breast cancer, systematic review, epidemiology, risk factors, regional effects

## Abstract

**Background:**

Numerous reviews of the epidemiology and risk factors for breast cancer have been published previously which heighted different directions of breast cancer.

**Aim:**

The present review examined the likelihood that incidence, prevalence, and particular risk factors might vary by geographic region and possibly by food and cultural practices as well.

**Methods:**

A systematic review (2017-2022) was conducted following Preferred Reporting Items for Systematic Reviews and Meta-analyses (PRISMA) guidelines, reporting on epidemiological and risk factor reports from different world regions. Medical Subject Heading (MeSH) terms: “Breast neoplasm” “AND” country terms such as “Pakistan/epidemiology”, “India/epidemiology”, “North America/epidemiology”, “South Africa/epidemiology” were used to retrieve 2068 articles from PubMed. After applying inclusion and exclusion terms, 49 papers were selected for systematic review.

**Results:**

Results of selected articles were summarized based on risk factors, world regions and study type. Risk factors were classified into five categories: demographic, genetic and lifestyle risk factors varied among countries. This review article covers a variety of topics, including regions, main findings, and associated risk factors such as genetic factors, and lifestyle. Several studies revealed that lifestyle choices including diet and exercise could affect a person’s chance of developing breast cancer. Breast cancer risk has also been linked to genetic variables, including DNA repair gene polymorphisms and mutations in the breast cancer gene (BRCA). It has been found that most of the genetic variability links to the population of Asia while the cause of breast cancer due to lifestyle modifications has been found in American and British people, indicating that demographic, genetic, and, lifestyle risk factors varied among countries.

**Conclusion:**

There are many risk factors for breast cancer, which vary in their importance depending on the world region. However, further investigation is required to better comprehend the particular causes of breast cancer in these areas as well as to create efficient prevention and treatment plans that cater to the local population.

## Introduction

Breast cancer (BC) is a major public health issue that affects women all over the world. It is the most often diagnosed cancer and the second biggest cause of cancer-related deaths among women globally (1). Breast cancer occurs at different rates around the world, with Western nations having greater incidence rates than Eastern nations. However, due to lifestyle changes, an increase in longevity, and the adoption of Westernized dietary practices, the prevalence of breast cancer is quickly rising in low- and middle-income countries (1). Several studies have shown that several factors, including age, race, and socioeconomic status, genetic factors like BRCA mutations, hormonal factors like age at menarche, parity, and age at first full-term pregnancy, breastfeeding, and lifestyle-related factors like diet, physical activity, alcohol use, and tobacco use are all associated with an increased risk of breast cancer (2).

Understanding and treating carcinoma of the breast on a global basis depends heavily on epidemiology. Breast cancer is the most prevalent kind of cancer in women globally, and its effects on people’s health as well as the general population cannot be overstated (3). We can gather and analyze data using epidemiology to better understand the distribution, risk factors, incidence, fatalities, and variations in the occurrence of breast cancer. The rate of incidence is significantly higher among old-aged women and the median age of breast cancer diagnosis was 63 years from year 2014-2018, which has, increased to 69 years during the years 2015-2019. However, the mortality rate has been reduced by 1.1% during 2013-2019; improving the average life span of the population due to the accessibility and availability of better healthcare facilities and timely diagnosis which has a profound impact on longevity factors. In Pakistan, the incidence of BC is increasing as compared to other Asian countries and the average life span is 67 years, which is less than the Western population. Since 2019, nearly 4 million patients with breast cancer have been living in the United States and the number of metastatic breast tumors revolts to one and a half million by 2021 (4, 5). The ratio of recurrence is almost 20-30% among the women who are treated or considered free of disease (6).

Globally women have been affected by several types of breast cancer, which are differentiated based on hormone levels, aetiology, clinical screening and availability of various treatment options. Commonly, invasive breast cancer types are classified into estrogen receptors (ER), progesterone receptors (PR) and human epidermal growth factor 2 (HER2). In Asia, the incidence of hormone-positive BC is relatively high as compared to other regions (7).

Determination of risk factors involved in the progression of breast cancer is especially important. Genetic factors such as gene mutations and family history are major threats to the development of cancer in first-degree relatives. Numerous biological processes, including histone modifications, polycomb/trithorax protein complexes, short non-coding or antisense RNAs, and DNA methylation, mediate epigenetic events. These various adjustments are intricately linked. The ability of genes to be expressed throughout typical stages of development is closely conditioned by epigenetic control (8). Histone deacetylases (HDACs) are a class of enzymes that play a critical role in the regulation of gene expression by modifying the acetylation status of histone proteins (9). Changes in the makeup of chromatin and the portability of DNA to DNA transcription factors can result from HDACs changing the acetylation status of histones, affecting the processes that lead to apoptosis (programmed cell death) and the cell cycle and altering the expression and function of hormone receptors such as the ER and PR, which may have an impact on hormone-dependent tumour growth. As a result, oncogenes may be activated or tumour suppressor genes may be silenced, accelerating the growth of cancer (10). Histone and non-histone proteins are acetylated by HDAC inhibitors (HDACi), which have an impact on gene expression, the advancement of the cell cycle, cell migration, terminal differentiation, and cell death. Understanding the anticancer mechanism(s) through which HDACi therapy drives differentiation in cancer may be crucial for understanding how GEF (guanine nucleotide exchange factor) protein regulation by HDAC inhibition influences cell differentiation (11). Age-related risks are closely related to the stage of menopause in women. Most women get affected with tumors at the post-menopausal stage (12). There is a strong association of breast density, obesity and hormonal imbalance with the incidence of breast cancer. Moreover, environmental and lifestyle risk factors, like toxic air pollution, occupational hazards, lack of physical activities, poor diet and smoking are contributing to the onset of BC (13). In leukemia and breast cells, HDAC expression and function are influenced by a variety of environmental variables. It has been demonstrated that environmental endocrine disruptors, change the expression and activity of the HDAC gene in breast cells (14). It is possible that altered HDAC activity plays a role in the emergence of leukemia, including acute myeloid leukemia (AML).

Chemicals known as endocrine disruptors prevent the endocrine system, which is in charge of producing and controlling hormones in the body, from operating normally. These substances have the potential to imitate or obstruct natural hormones, resulting in hormonal imbalances and possibly harmful consequences on health (15). Increased estrogen activity may result from exposure to endocrine disruptors, which may then promote the development of hormone-sensitive breast cancer cells. Certain endocrine-disrupting substances, especially bisphenol A (BPA) and phthalates, have been linked in studies to an increased risk of breast cancer. During the last several decades, there has been an increase in the prevalence of breast cancer worldwide. While many causes have contributed to this increase, endocrine disruptors are one cause for concern (16). Understanding the global epidemiology of breast cancer based on risk factors is essential for developing effective prevention and treatment strategies tailored to local populations. Therefore, this systematic review aims to evaluate the available evidence on the global epidemiology of breast cancer based on risk factors by systematically collecting recent published literature (2017-2022).

## Methodology

### Search strategy

**Figure 1:**
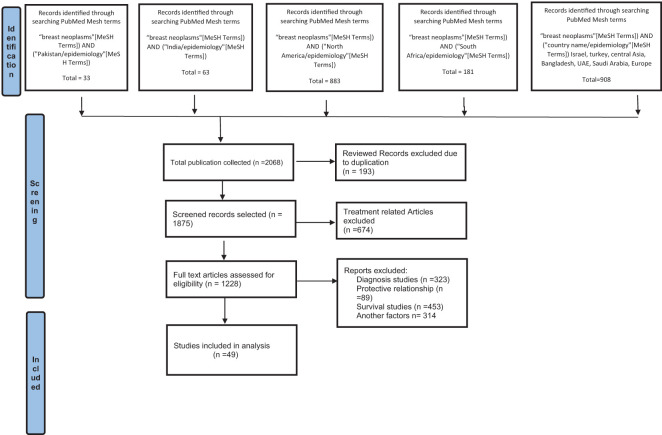
PRISMA flow diagram of study selection.

Systematic review of the literature utilizing PubMed was performed according to PRISMA 2020 guidelines (17) (**Figure 1**; PRISMA flow diagram), as used in our previous systematic reviews (18, 19). PubMed is frequently suggested in guidelines for systematic reviews and covers a sizable amount of the literature pertinent to our research question. Furthermore, one of the unique features of PubMed is the Medical Subject heading (MeSH) terms, which are employed in PubMed for systematic review literature searches because they raise the standard and dependability of search results. The National Library of Medicine established MeSH words as a regulated vocabulary for indexing and annotating papers, and PubMed is a biological database that incorporates citations to pertinent material (20). By including both index terms from standardized terminologies like MeSH and free-text terms, using MeSH terms enables researchers to conduct more thorough searches (21). MeSH words offer a standardized approach to represent concepts and themes, guaranteeing that all pertinent articles are included in the search and assisting in the identification of pertinent articles (22). By enabling researchers to insert more precise terms associated with the study question, they also aid in the refinement of search results (23).

All publications were retrieved from PubMed in September 2022, with Medical Subject Heading (Mesh) Terms; a new and thoroughly revised version of lists of subject headings compiled by the National Library of Medicine (NLM) for its bibliographies and cataloging. The Mesh term “Breast neoplasm” was used with the Boolean operator “AND” and other related Mesh Terms related to regions/country names and “Epidemiology” to search all the records available from 2017 to 2022.

### Study selection

A detailed list of retrieved articles related to BC epidemiology based on risk factors was collected for quantitative analysis. The initial screening was based on the title and abstract, while the final inclusion was based on full texts where available. EndNote software was used to combine and sort out duplicated articles based on the inclusion and exclusion criteria. All authors reviewed the retrieved articles and included only those articles, which were fulfilling the following conditions.

#### Inclusion criteria

Full-text articles published in PubMed Indexed journals, indexed with Mesh Terms as stated above.

#### Exclusion criteria

Abstracts, short commentaries, and studies focusing on treatment, and/or in languages other than English were excluded. Systematic reviews and letters to the editors were not included in this review. Qualitative studies regarding treatment therapies, survival rates, and diagnostic irregularities were excluded because of their inappropriate focus on the aim of our review.

### Data extraction

The first authors of this manuscript independently performed data extraction. All disagreements were discussed and resolved by all other authors in this study. The following data taken from each article was entered into a spreadsheet: Study reference, year published, study design, study region and risk factors.

### Quality assessment

Three investigators independently rate the quality of included study as good, fair or poor. Final ratings were determined by consensus among all reviewers, only those studies rated as good or fair were included.

## Results

### Study selection

An extensive search was conducted in PubMed using advanced search strategies to identify articles related to breast neoplasms in different regions. The search terms utilized were “breast neoplasm” and “Pakistan/epidemiology”, which resulted in 33 articles being extracted. After a rigorous process of inclusion and exclusion criteria, 11 articles on prevalence studies were selected for further analysis.

Similarly, the search terms “breast neoplasms” and “India/epidemiology” were used, resulting in 63 articles being extracted. Out of these, 14 articles were deemed suitable for epidemiological studies after applying the selection criteria. The search terms “breast neoplasms” and “North America/epidemiology” produced a total of 883 articles, and 11 of these were selected for the study. The search terms “breast neoplasms” and “South Africa/epidemiology” produced 181 articles, with 6 being selected. Finally, the search terms “breast neoplasms” and “Israel/Turkey/Central Asia/Bangladesh/UAE/Saudi Arabia/Europe/Epidemiology” produced 908 articles, and 21 were selected for the study.

The above results demonstrate the comprehensive nature of the literature search and thorough application of the inclusion and exclusion criteria.

### Study characteristics

An initial search in PubMed utilizing MeSH terms (described above) resulted in the extraction of 2068 articles. Duplicate articles (n=193) were removed, leaving 1875 articles for further review. The remaining articles were evaluated by examining their titles and abstracts, and after applying the selection criteria, 49 studies were included in the present review, as shown in the PRISMA flow chart (**Figure 1**). The studies selected are summarized in **Table 1**, which highlights the reference, design, risk factors, sample size and type of the studies included in this systematic review. **Table 2** presents the proportion of risk factors in various regions of the World.

**Table 1:** Main results, risk factors and study design of studies associated with breast cancer incidence in various regions of the world.

Reference	Main Results/Findings and Risk Factors	Sample size	Study type/design
	Asia		
(24)	Breast cancer incidence rates were higher in Asian Indian and Pakistani Americans (AIPA) than in non-Hispanic white Americans (NHW). Family history of breast cancer, reproductive factors	4900 AIPA and 482 250 NHW	Surveillance, Epidemiology and End Results-based study
(25)	Breast cancer was more common among postmenopausal women who had early menarche, late menopause, and a positive family history of breast cancer	326 women	Cross-control study
(26)	Breast density was positively associated with age, body mass index (BMI), and parity, and negatively associated with smoking and oral contraceptive use	477 women	Cross-sectional study
(27)	Breast cancer incidence was projected to increase over time, particularly among women aged 50 years and older.	9771 registered diagnosed cases	Time-trend analysis
(28)	Metaplastic breast carcinoma was associated with worse survival outcomes compared to invasive ductal carcinoma (Histological type of cancer)	42 patients	Retrospective closed Cohort study
(29)	Epstein-Barr virus (EBV), human papillomavirus (HPV), and mouse mammary tumor virus (MMTV) were detected in breast cancer tissue samples, suggesting a possible etiological role of these viruses in breast cancer	tissue biopsies (n = 250)	Case-control study
(30)	P53 overexpression was associated with hormone receptor status and triple-negative breast carcinoma	91 patients	Retrospective study
(31)	Younger breast cancer patients (<40 years old) had more advanced cancer at diagnosis and worse survival outcomes compared to older patients (Age)	1,334 patients	Retrospective study
(32)	Transforming growth factor β1 (TGFβ1) gene polymorphism (T29C) was associated with an increased risk of breast cancer	150 subjects, 80 cases and 70 healthy controls	Case-control study
(33)	The prevalence of BRCA1/2 mutations was higher in Indian breast and/or ovarian cancer patients than non-BRCA mutations	1010 patients	Multi-gene panel screening
(34)	Delays in diagnosis and treatment of breast cancer were associated with lack of knowledge about breast cancer symptoms and risk factors, as well as poor referral systems	269 breast cancer patients	Mixed-methods study
(35)	Obesity was associated with increased oxidative stress in breast cancer patients	30 patients women, 30 healthy control	Cross-sectional study
(36)	Lack of knowledge about breast cancer symptoms and risk factors was common among women in a low socio-economic area of Mumbai	480 women	Community-based study
(37)	Low serum levels of 25-hydroxyvitamin D were associated with an increased risk of breast cancer in Indian women	297 subjects	Case-control study
(38)	The prevalence of breast cancer screening was low among women aged 30-49 years in India, and was associated with higher education, urban residence, and wealth.	336,777 women aged 30-49 years	Secondary data analysis
(39)	Air pollution emissions are associated with a higher incidence and prevalence of breast cancer in the Aktobe region of western Kazakhstan		Retrospective study
(40)	Genetic polymorphisms in the DNA repair genes XRCC1 and XRCC3 may be associated with breast cancer susceptibility in Bangladeshi women	121 breast cancer patients and 133 healthy controls	Case-control study
(41)	Gene-positive breast cancer in UAE had an earlier age of onset, higher rates of bilateral tumors, and lower rates of lymph node involvement compared to gene-negative tumors	309 patients	Retrospective study
(42)	Sedentary lifestyle and unhealthy dietary habits were associated with an increased risk of breast cancer among women attending an oncology day treatment center in Turkey	65 diseased women, 65 healthy women	Case control study
(43)	Younger age at diagnosis was associated with worse outcomes in breast cancer patients, particularly those aged 25 years or younger	137 patients	Histopathological and clinical study
(44)	HER2 over-expressed breast cancer was found to be more aggressive and associated with poorer prognosis in Saudi Arabian women	1867 patients	Retrospective study
(45)	Triple-negative breast cancer was the most common subtype among Saudi Arabian women and was associated with younger age at diagnosis	270 female patients	multi-centric, Cross-sectional study
(46)	Breast cancer patients in Botswana presented with a more advanced stage of disease and had lower survival rates compared to patients in South Africa and the United States (Late presentation)	Botswana (*n* = 384, 2011-2015), South Africa (*n* = 475, 2016-2017), and the US (*n* = 361,353, 2011-2012)	Retrospective study
(47)	Hormone receptor-positive tumors were the most common subtype of breast cancer in Rwanda, and were more commonly diagnosed at advanced stages	138 patients	Retrospective study
	Africa		
(48)	Inherited breast cancer is a significant issue among Nigerian women, and the BRCA1/2 mutations account for a large proportion of inherited cases	1,136 women, 997 women without cancer	Case-control study
(49)	The prevalence of inherited mutations in breast cancer predisposition genes among women in Uganda and Cameroon is relatively low, with BRCA1/2 mutations being the most common	196 cases and 185 controls	A multigene sequencing panel
(50)	Low vitamin D status and VDR genetic polymorphisms are associated with an increased risk of breast cancer in Ethiopian women	392 female breast cancer patients and 193 controls	Case-control study
	America		
(51)	Hair dye and chemical straightener use are associated with an increased risk of breast cancer in black women, but not in white women	participants (*n* = 46,709), women ages 35–74	Prospective cohort study
(52)	A healthful plant-based diet is associated with a lower risk of breast cancer, whereas an unhealthful plant-based diet is associated with a higher risk of breast cancer	76,690 women from the Nurses’ Health Study (NHS, 1984–2016) and 93,295 women from the NHSII (1991–2017).	Prospective cohort study
(53)	Weight loss is associated with a reduced risk of breast cancer in postmenopausal women (Obesity)	Postmenopausal women (n = 61,335)	Observational study
(54)	Sugar-sweetened soda consumption is associated with an increased risk of breast cancer mortality	927 breast cancer cases	Western New York Exposures and Breast Cancer Study
(55)	Smoking is associated with an increased risk of breast cancer, particularly in hormone receptor-positive tumors, in African American women	67 313 women, 45–75 years of age	Multiethnic Cohort (MEC) study
(56)	Blood levels of endocrine-disrupting metals are associated with an increased risk of breast cancer in American women.	9260 women aged ≥ 20 years	multivariate logistic regression models
(57)	Obesity and diabetes are independently associated with an increased incidence of breast cancer in Louisiana.	Luminal A (*n*=1,584), TNBC 364 Luminal B 232 and HER2 + 115	retrospective case-control study
(58)	Variations in TNFα, PPARγ, and IRS-1 genes are associated with survival in breast cancer patients.	breast cancer between 1995 and 1999	Prospective cohort study
(59)	Certain occupations and industries, such as healthcare and the service sector, are associated with an increased risk of breast cancer in both women and men	Women 17 865 and Men 492	Occupational Disease Surveillance System cohort
(60)	Exposure to ambient air emissions of polycyclic aromatic hydrocarbons is associated with an increased incidence of breast cancer in American women	N/A	Ecological study
	Europe		
(61)	Joint tobacco smoking and alcohol intake increase cancer risk	19,898 women	Questionnaires
(62)	Long-term consumption of non-fermented and fermented dairy products is not associated with breast cancer risk	33,780 women	Population-based prospective cohort study
(63)	Occupational exposure to organic solvents, including ethanol, is associated with increased breast cancer risk	38,375 breast cancer cases and 191,875 controls	population-based nested case–control study
(64)	Benign breast diseases are associated with age, hormonal factors, and family history of breast cancer	61 617 women	cohort study
(65)	Thyroid gland diseases are associated with increased breast cancer risk	7408 women	retrospective case–control study
(66)	Employment in certain industries is associated with increased breast cancer risk	845 women	population-based case-control study
(67)	Adherence to healthy lifestyle behaviors is associated with reduced breast cancer risk, and this association is stronger in women without a genetic predisposition to breast cancer	146326 women	COX proportional hazard regression model
(68)	Occupational heat exposure is associated with increased breast cancer risk	1,738 breast cancer cases and 1,910 controls	Case-control study
(69)	Smoking is associated with increased breast cancer risk	102,927 women	Generations Study cohort
	Israel		
(70)	Breast cancer incidence is increasing among younger women (Age)	34,251 women	Cross-sectional study
(71)	Inherited predisposition to breast and ovarian cancer is observed in non-Jewish populations in Israel (Genetic factors)	68 cases	Population study
(72)	Cumulative mammographic density is positively associated with age-specific incidence of breast cancer	200 women	Cohort study
(73)	Passive smoking is associated with increased breast cancer risk in women with NAT2 polymorphism	137 breast cancer patients 274 population-based controls	population-based case-control study

**Table 2:** Risk factors associated with breast cancer in different regions of the world and populations.

Reference	Region	Age	Obesity	Breast Density	Genetic Mutation /Family History	History of Cancer	Hormonal Imbalance	Pregnancy	Lifestyle Factors\diet	Smoking/Alcohol	Drug Abuse	Infections/Diseases	Air Pollution/Occupation
Asia		Demographic			Genetic		Hormonal Imbalance		Lifestyle			Others	
**(** 24 **)**	Asian Indian, Pakistani Americans	+				+							
**(** 25 **)**	Southern Punjab, Pakistan	+	+			+	+		+				
**(** 27 **)**	Karachi, Pakistan	+		+									
**(** 26 **)**	Karachi, Pakistan	+		+									
**(** 28 **)**	Karachi, Pakistan		+		+					+	+		
**(** 29 **)**	Pakistan											+	
**(** 30 **)**	Lahore, Pakistan				+		+						
**(** 31 **)**	Karachi, Pakistan	+											
**(** 32 **)**	Rawalpindi, Pakistan				+								
**(** 33 **)**	India				+								
**(** 34 **)**	North East India	+					+						
**(** 35 **)**	India		+										
**(** 36 **)**	Mumbai, India		+				+	+					
**(** 38 **)**	India	+	+					+	+				
**(** 39 **)**	Western Kazakhstan												+
**(** 40 **)**	Bangladesh				+								
**(** 41 **)**	UAE	+				+						+	
**(** 42 **)**	Turkey								+				
**(** 43 **)**		+											
**(** 44 **)**	Saudi Arabia				+								
**(** 45 **)**		+											
**(** 46 **)**	South Africa	+					+		+				
**(** 47 **)**	Rwanda	+					+						
**(** 48 **)**	Nigeria				+								
**(** 50 **)**	Ethiopia								+				
**(** 51 **)**	North America								+				
**(** 52 **)**									+				
**(** 53 **)**			+										
**(** 54 **)**									+				
**(** 55 **)**										+			
**(** 74 **)**									+				
**(** 56 **)**													+
**(** 57 **)**			+									+	
**(** 58 **)**					+								
**(** 59 **)**													+
**(** 60 **)**													+
**(** 61 **)**	Denmark								+	+			
**(** 62 **)**	Sweden								+				
**(** 63 **)**	Denmark									+			+
**(** 64 **)**	Sweden	+			+		+						
**(** 65 **)**	Germany											+	
**(** 66 **)**	UK												+
**(** 67 **)**	Spain												+
**(** 75 **)**	UK												+
**(** 69 **)**	Poland									+			
**(** 70 **)**		+					+						
**(** 71 **)**					+								
**(** 72 **)**				+									
**(** 73 **)**					+					+			

**Tables 1**, **2** present various studies conducted on breast cancer incidence and risk factors in different regions of the world. The findings were discussed based on the study design and risk factors and geographical region.

In the Asian region, studies have found that breast cancer incidence rates are higher in Asian Indian and Pakistani Americans than in non-Hispanic white Americans (24). Risk factors identified include a family history of breast cancer, early menarche, late menopause, positive family history, and obesity (25). Viral infections, genetic mutations, lack of knowledge about breast cancer symptoms and risk factors, and low vitamin D levels are also associated with an increased risk of breast cancer (26–32). The prevalence of BRCA1/2 mutations is higher in Indian breast and/or ovarian cancer patients, and delays in the diagnosis and treatment of breast cancer are associated with poor referral systems (33).

In Africa, inherited mutations in the BRCA1/2 genes have been found to be a significant issue among Nigerian women (48). Low vitamin D status and VDR genetic polymorphisms are associated with an increased risk of breast cancer in Ethiopian women (49, 50).

In the USA, studies also identified risk factors such as use of hair dye and chemical straightener (51), unhealthy plant-based diet (52), obesity and diabetes (53, 57), sugar-sweetened soda (54), certain genetic variations (58), occupational exposure to organic solvents (59), smoking (55) and endocrine-disrupting metals (56) that are associated with increased breast cancer incidence and mortality.

Certain occupations and industries, such as healthcare and the service sector, are also associated with increased risk (60). Weight loss is associated with a reduced risk of breast cancer in postmenopausal women. Studies have also identified genetic variations associated with survival in breast cancer patients.

In Europe, studies have found that joint tobacco smoking and alcohol intake (61), occupational exposure to organic solvents and ambient air emissions of polycyclic aromatic hydrocarbons (63), age, hormonal factors, and family history of breast cancer (64), thyroid gland diseases (65), and employment in certain industries (66) are associated with an increased risk of breast cancer. Adherence to healthy lifestyle behaviors is associated with reduced breast cancer risk, and this association is stronger in women without a genetic predisposition to breast cancer (67). In addition, smoking and alcohol intake increases cancer risk (69, 75).

In Israel, studies have found that breast cancer incidence is increasing among younger women (70). Genetic factors, including inherited predisposition to breast and ovarian cancer (71, 73), and mammographic density (72) are also associated with increased breast cancer risk.

**Figure 2:**
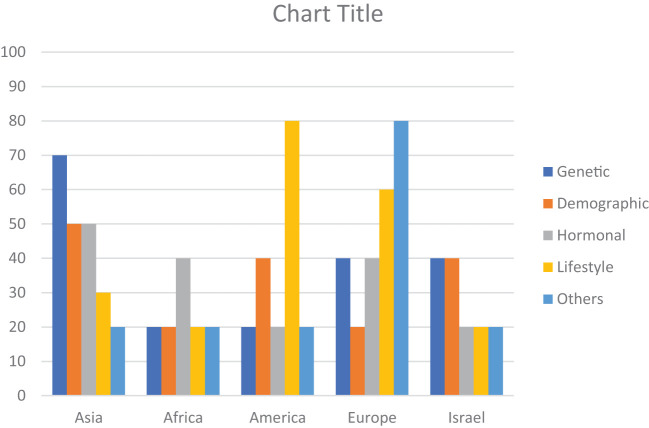
Region-wise percentage prevalence of risk factors. Demographic (Age, Obesity, Breast Density), Genetic (Genetic Mutation, Family History, History of Cancer), Hormonal (Hormonal Imbalance, Pregnancy), Lifestyle (Lifestyle Factors, diet, Smoking/Alcohol, Drug Abuse), Other (Infections/Diseases, Air Pollution/Occupation).

The risk factors for breast cancer are subdivided into demographic, genetic, hormonal, lifestyle, and other categories as shown in **Table 2**. The percentage prevalence by area is also shown in **Figure 2**, which demonstrates that genetic and societal variables are the most prevalent risk factors for breast cancer in Asia, with a prevalence of 70 and 50, respectively. Additionally important are lifestyle factors, which have a prevalence of 50 and 30, respectively, and hormonal aspects.

With a frequency of 40, hormonal variables are the most common risk factor for breast cancer in Africa. With a prevalence of 20 each, genetic, demographic, and other (Air pollution, Oxidative stress, Infections/Diseases, Occupations) factors are also significant. In America, lifestyle factors are the most significant risk factor for breast cancer, with a prevalence of 80. Genetic, demographic, and hormonal factors also contribute, with a prevalence ranging from 20 to 40.

In Europe, other factors such as oxidative stress, infections, diseases, and occupations have the highest prevalence, with a prevalence of 80. Hormonal, genetic, demographic, and lifestyle factors also play a role, with a prevalence ranging from 20 to 60.

In Israel, genetic and demographic factors have an equal prevalence of 40, followed by hormonal and lifestyle factors with a prevalence of 20 each. Other factors have a prevalence of 20.

## Discussion

Patients with breast cancer have multiple risk factors associated with their disease (76). Depending on the characteristics of specific geographical regions, certain risk factors either modifiable or non-modifiable have variable influences on the health of women. Early identification of modifiable factors helps develop strategies to reduce the incidence of Breast cancer whereas other factors such as age, gender, and family history are not in an individual’s control to avoid breast cancer risk (77). Hormone positive breast tumor is quite common among Asian women. **Figure 2** shows that approximately 50% of women have imbalanced hormonal levels, which increases the chances of BC development whereas in Europe and Africa, the estimated prevalence of BC due to hormonal abnormalities is 40%. Various risk factors contribute to the progression of breast tumors at various levels. All regions discussed in this review showed variable data on individual factors associated with the prevalence of BC all over the world. Presence of mutant genes (BRCA1 and BRCA2) can increase the incidence of BC up to 80% of women populations as compared to non-mutant genes (78). Few mutant genes (CHEK2, PTEN, CGH1, STK1 and PALB2) do not impose much influence on the occurrence of BC. Despite this genetic variability, a few genes (RAD52, OCT4, FASL, IGFIR, APE1, BARD1, IL4, and IL21) pose a protective impact and decrease the risk of developing BC. Chances of BC are significantly high if the patient has a positive BC family history even in men. Overall, the prevalence of BC in males is quite low but family history increases the risk in males as well. This trend is confirmed in various studies conducted in different regions of the world. We discussed association of various risk factors with specific geographical regions in the following sections.

## Asia

A person’s demographic group or a particular subset of the population can have an impact on the occurrence, distribution, assessment, and management of breast cancer through certain characteristics. Demographic considerations can shed light on the patterns and trends in the incidence of breast cancer in various communities (79). Age, weight, and breast density are highly correlated with the incidence of BC (80). According to a study in Pakistani Asian women, younger females are more affected by BC and its prevalence increased from 70% to 130% among females aged 30 to 34 years and among the age group 50-64 years, the percentage prevalence increased from 23.1% to 60.7% (26) (**Table 1**). Particularly, the incidence of metastatic BC and high-grade BC in young females has escalated in the past few years. The frequency, grade at being diagnosed, and available treatments for breast cancer can all be influenced by socioeconomic factors like income, education, and access to the hospital (81). Due to the socioeconomic problems in Asian countries, early diagnosis and timely screening is not accessible (31). People from rural areas have faulty beliefs and feel hesitation at the time of mammographic inspection (82). This reluctant behavior is a major reason for the increased incidence of BC at a young age. According to certain studies, married women might receive a better prognosis than single or divorced women (83). In adolescents, 86% of patients are diagnosed with invasive ductal carcinoma, 16.8% have luminal A and 30.5% patients have luminal B cancer. 30% of patients were affected by HER2+ whereas only 15% showed diagnosis with triple negative BC (32). Late diagnosis in developing countries drastically increased the progression to late stage tumor. In a recent study, Prevalence of stage III cancer was 62% whereas 24.8% patients were diagnosed with stage II cancer (47).

Breast cancer risk is heavily influenced by hereditary variables, and several genetic variants are known to dramatically enhance the risk of developing the illness. BRCA1 and BRCA2 gene mutations are the most well-known genetic changes linked to breast cancer. These genes are crucial for preserving the stability of the genetic material in the cell since they are involved in mending damaged DNA. The chance of developing breast and ovarian cancers is considerably increased by inheriting a deleterious mutation in either the BRCA1 or BRCA2 gene (84). An association has been observed between genetic mutations and the risk of BC. In Asia, Approximately 70% of patients have genetic polymorphism, DNA repair, overexpression of p53, presence of BRCA1 and BRCA2, and other hereditary characteristics (**Figure 2**), whereas the risk of BC in other regions due to genetic mutation is comparatively low. High occurrence of breast cancer due to the genetic mutation in BRCA1 and BRCA2 genes in Asian women is directly related to first-degree relatives (85).

## Africa

A complex interaction of factors, including genetics, way of life, socioeconomic circumstances, healthcare infrastructure, and cultural beliefs, characterizes the epidemiology of breast cancer in Africa. The female hormones progesterone and estrogen can affect the development of breast tissue and cells, and both their levels and activities are linked to an increased risk of breast cancer. A hormone called estrogen promotes the growth and upkeep of female reproductive tissues. High amounts of estrogen or continuous exposure to estrogen can raise the likelihood of breast cancer because it can encourage cell development in the breast. Imbalance of hormonal profile in the female population is the major risk factor for developing BC (86). Proliferation of cancer cells can be aggressive if estrogen and progesterone levels are not up to the mark. Breast cancer risk has been linked to long-term usage of combination hormone replacement therapy (estrogen and progestin) during menopause (87). Premenopausal and postmenopausal stages are highly linked with the occurrence of BC (88). Existing research on breast cancer in Africa is characterized by a limited collection of studies. According to the limited collections of studies conducted in Africa have been shown that 40% involvement of hormonal factors in the prevalence of BC. In addition, other factors; including Infections/Diseases, Air Pollution/Occupation, have been found to equally contribute to the occurrence of breast cancer within the African population. However, it is important to note that the lack of resources in many African regions poses significant challenges to collecting precise and comprehensive data. To gain a more comprehensive understanding of the distinct patterns of breast cancer in different African locations and to tailor therapeutic interventions accordingly, a more extensive and rigorous research effort is warranted.

## America

Susceptibility of inherited mutations in America and Africa is modest however; nearly one-third of the female population of Europe and Israel is under threat of BC progression due to genetic mutations (**Figure 2**). If a person contains dangerous mutations in breast cancer-related genes, genetic testing can reveal this. For the evaluation of risks, prevention tactics, and screening advice, this information may be essential (89).

Lifestyle modifications impart beneficial effects on women’s health. Women who are exposed to smoking, containing toxic aromatic compounds and consuming alcohol, are more prone to developing breast cancers (61, 63). Physical inactivity on a regular basis is linked to an increased risk of breast cancer. It has been demonstrated that regular physical activity lowers the incidence of breast cancer (90). Most of the population of America and Europe have a sedentary lifestyle and unhealthy eating habits (51) and the affected population with BC is 80% and 60% respectively (**Figure 2**). Prevalence of lifestyle risk factors in other geographical regions is very low which may involve certain social and ethical problems (91).

Based on race and ethnic origin, the incidence rate is higher in black women as compared to white women (92). A recent surveillance and epidemiology study demonstrated that Asian Indian and Pakistani women who reside in the United States have a high degree of BC incidence ratio as compared to non-Hispanic white women (24). Based on age, young and late menopausal age are most affected by this life-threatening disease because of imbalanced hormonal profiles. Other factors including late pregnancy, use of contraceptive pills and hormonal therapies for conception alter the normal levels of estrogen and progesterone, which are the main hormones involved in the growth of BC.

Obesity is linked with majority of chronic diseases including breast carcinoma. A higher risk is observed in menopausal women who are obese and have a sedentary lifestyle as compared to the females having normal BMI (93). Unhealthy eating habits, consumption of Trans fats and dawn-to-dusk working hours affect the normal physiological processes of our body and increase the risk of developing cancer. Physical activities like walking and aerobic exercises help to reduce the threat of BC to a greater extent (94).

## Europe

Air pollution, drug abuse and infections have a deleterious influence on the European population that affected 80% of the population (**Figure 2**). Occupational hazards thrust including exposure to organic solvents and fumes of dangerous gases are more prominent causes of health problems in Europe (75). Moreover, noise pollution is also a crucial risk factor that is associated with the etiology of BC (75).

In addition to physical workouts, a healthy diet and consumption of essential vitamins reduce the risk of BC. Several studies have shown that intake of vitamin D with treatment has positive outcomes in cancer patients thus slowing the progression of the disease whereas its deficiency can increase the BC risk (95). Moreover, consumption of alcohol and smoking is linked with a higher incidence of BC and it is evident by various studies (69). Occupational toxic exposure and air pollution are also contributing factors in the occurrence of BC all over the world because of global climate alterations (96).

Quercetin (QCT), a flavonoid derived from many fruits and vegetables, is endowed with manifold biological properties, such as the ability to elicit a strong inhibitory effect on the growth of several tumor cell lines (97). Quercetin may aid in preventing DNA deterioration in cells and thwarting the formation of cancer cells by lowering oxidative stress (98). The BRCA genes’ expression may be affected by quercetin, perhaps improving their capacity for DNA repair (99). Research has been done on quercetin’s potential to lessen breast density, which could, in turn, reduce the risk of breast cancer. According to certain studies, quercetin can modify estrogen metabolism and affect hormone levels, which may affect the composition and density of breast tissue (100). QCT has been proposed as an auxiliary molecule when combined therapy, when given along with many chemotherapeutic medications, such as topotecan, cisplatin, and sorafenib, in the treatment of various malignancies (97). According to this review, genetic and hormonal risk factors contributed 40% toward prevalence of BC in women but lifestyle modification factors 60% associated with BC.

Obesity and breast cancer have a complicated and varied association. Insulin resistance and persistent low-grade inflammation are both linked to obesity. These elements can foster a body environment that is conducive to the growth of breast cancer (101). Due to increasing breast density, people may find it harder to identify breast tumors or abnormalities, which can delay diagnosis and treatment. Compared to non-obese patients, obese breast cancer patients are more likely to have a cancer recurrence and are at a higher risk of dying from the disease (102). Obesity has an inverse relation with menopause age that contributes to the development of Breast cancer (103). An observational study conducted in Europe and America explained that the risk of BC due to obesity was lower at premenopausal age as compared to postmenopausal age (53).

## Israel

In Israel, breast cancer is by far the most prevalent type of cancer among women. Because of variables like longer life expectancies, altered reproductive habits, and lifestyle choices, prevalence rates have been continuously increasing. The mortality rate has been declining, nevertheless, in part because of breakthroughs in therapy, early detection, and screening techniques. Women between the ages of 50 and 74 can receive mammograms through Israel’s national breast cancer screening program. The goal of this initiative is to identify breast cancer early, when it can be treated more successfully. The decreasing mortality rates have been attributed to routine screening and early diagnosis (104). Particularly among Ashkenazi Jewish women, Israel’s population is distinct in that some genetic variants are relatively common. This population has a greater prevalence of BRCA1 and BRCA2 gene mutations, which increases the chance of getting breast and ovarian cancer and reported elevated carrier frequency of 0.9% in the Ashkenazi Jewish population, a specific BRCA1 mutation known as 185delAG is also occasionally seen in non-Jewish patients with a distinct haplotype (105). Breast density is another factor associated with the incidence of BC as females with dense breasts are at a higher chance of developing BC as compared to those with less dense breasts (72). Early diagnosis by mammography is significantly difficult in dense breasts, which leads to the progression of late-stage BC. On the other hand, certain diseases such as diabetes, hypertension, insulin intolerance, multiple sclerosis and polycystic ovary syndrome (PCOS) also increase the risk of BC (106). Our data showed that in Israel demographic and genetic factors are predominant (40%) among the population as compared to other risk factors, which associated with occurrence of BC only 20% (**Figure 2**). For the most up-to-date details about breast cancer epidemiology in Israel, it is crucial to study the most recent sources, including Israeli health authorities, cancer registries, and research organizations.

## Conclusion

This paper has reviewed studies of incidence, prevalence, and risk factors for breast cancer in India, Pakistan, Kazakhstan, Turkey, USA, Europe and the United Arab Emirates. The evidence shows that diet, obesity, and genetic factors, such as BRCA mutations and DNA repair gene polymorphisms vary from region to region. These findings emphasize the need to be aware of the particularities of each region of the world with respect to breast cancer risk. This will facilitate early detection and improve prognosis. Our study provides valuable insights into the epidemiology, risk factors, and outcomes of breast cancer in various populations and highlights the need for further research and intervention efforts to reduce the burden of breast cancer in these regions.

## Study limitations

Not all world regions were included and study methodologies varied. In addition, the traditions, customs, and genetic backgrounds of the residents of different geographic regions is only superficially known. Thus, more specific research is needed that specifically target distinct populations or examine particular risk factors in order to enhance the comprehensiveness and accuracy of findings.

## Data availability statement

The original contributions presented in the study are included in the article/supplementary materials, further inquiries can be directed to the corresponding author/s.

## Author contributions

AR played a major role in the literature search, collecting and screening of abstracts, full-text articles, data extraction and quality assessment of the included studies and prepare the first draft of the manuscript, AK provide the Idea and design for systematic review, supervised and guided AR in data collection and reporting and review and revised the manuscript. FA and ZA: screening of abstracts, full-text articles, data extraction and quality assessment of the included studies and revision of the manuscript. MA, MK, MS, RA: critical revision of manuscript and resolution of discrepancies. All authors made significant intellectual contributions and approved the final version of the manuscript.
